# How to support staff deploying on overseas humanitarian work: a qualitative analysis of responder views about the 2014/15 West African Ebola outbreak

**DOI:** 10.3402/ejpt.v7.30933

**Published:** 2016-11-17

**Authors:** Gideon James Rubin, Sarah Harper, Paolo Diaz Williams, Sanna Öström, Samantha Bredbere, Richard Amlôt, Neil Greenberg

**Affiliations:** 1 Department of Psychological Medicine, Weston Education Centre, King’s College London, London, UK; 2 Emergency Response Department Science and Technology, Health Protection and Medical Directorate, Public Health England, Salisbury, UK

**Keywords:** Infectious diseases, humanitarian response, psychological support, training, wellbeing, distress

## Abstract

**Background:**

Responding to health crises overseas can be both rewarding and distressing for staff involved.

**Objective:**

We interviewed UK staff involved in the 2014/15 Ebola response to identify experiences that positively or negatively affected them.

**Method:**

We conducted qualitative telephone interviews with 30 Public Health England (PHE) staff and 21 non-governmental organisation (NGO) staff who had deployed to West Africa.

**Results:**

The main motivations for deploying were for moral reasons and personal development. Families were largely supportive of deployment, although family tension was apparent. Pre-deployment training was largely viewed positively. Common stressors included dealing with death and suffering as well as concerns about contagion, while uplifting aspects included seeing patients improve and receiving thanks from community members. Communications with home were largely satisfactory, although participants commonly self-censored their communication. Inter-organisational tensions caused stress, particularly for PHE staff hosted by NGOs. After deployment, loss of motivation and being avoided by friends and family were common.

**Conclusion:**

Highlighting the personal benefits arising from deployments, as well as their moral value, may help to increase volunteering. Efforts to improve the support given to responders should focus on identifying how to better support families, preparing all staff members for dealing with death and the risk of contagion, providing opportunities for staff to more frequently experience the uplifting aspects of deployment, resolving inter-organisational difficulties, and educating others about the low risk posed by responders on their return.

**Highlights of the article:**

- We interviewed 51 medical and laboratory staff sent to West Africa during the 2014 Ebola outbreak, to understand what factors were stressful or uplifting.
- Common stressors included dealing with death and suffering as well as concerns about contagion. Uplifting aspects included seeing patients improve and receiving thanks from community members. After deployment, loss of motivation and being avoided by friends and family were common.
- Preparation and support for staff should consider these issues.

On 23rd March 2014, the World Health Organization confirmed that an outbreak of the Ebola virus had occurred in Guinea, West Africa. The outbreak subsequently spread to Liberia and Sierra Leone. By April 2015, the death toll was estimated at over 11,000 (Centers for Disease Control and Prevention, 2015). As part of the international public health response to the outbreak, hundreds of aid workers, medical personnel, and laboratory staff were deployed to West Africa. The UK’s contribution included National Health Service (NHS) staff and charity workers, who largely assisted with the clinical treatment of patients within dedicated Ebola treatment centres, and Public Health England (PHE) staff, who were broadly responsible for setting up laboratories and conducting diagnostic tests in order to identify people with Ebola. While many of the NHS and charity workers who went to West Africa had experience of working in previous humanitarian crises, this was the first time that PHE had deployed large numbers of laboratory staff to support an overseas crisis, many of whom had not worked in such circumstances before.

Emergency response work can be rewarding, distressing and affect mental health (Bakhshi et al., 2014; Clukey, 2010; Perrin et al., 2007; Soliman, Lingle, & Raymond, 1998; Strohmeier & Scholte, 2015; Thoresen, Tonnessen, Lindgaard, Andreassen, & Weisaeth, 2009; Yokoyama et al., 2014). As overseas deployments for health crises become more common, it is important to understand what factors contribute to positive and negative outcomes for the staff involved (Greenberg, Wessely, & Wykes, 2015). A recent review identified several factors that affect the wellbeing of humanitarian workers (Brooks et al., 2015). However, there has been little research on responders to an infectious disease outbreak which poses a direct threat to workers and which may cause higher levels of distress (Koh, Hegney, & Drury, 2011; Maunder, 2004; Styra et al., 2008). In this qualitative study, we explored the issues that affected UK staff involved in the 2014/15 Ebola response to identify ways of encouraging volunteering and improve support provision for future incidents.

## Method

### Design

We conducted interviews from December 2014 to February 2015 with PHE or non-governmental organisation (NGO) staff who had returned from West Africa. For PHE, we randomly contacted staff from a database of deployed personnel. For NGO staff, two charities sent emails about our study to all their returnees. Leaflets about the study were also included in packs given to all returnees by PHE.

### Ethics

The Psychiatry, Nursing and Midwifery Research Ethics Subcommittee at King’s College London approved the study (PNM/14/15-30). Participants were provided with an information sheet and consent form.

### Procedure

Interviews were conducted via telephone, recorded, and fully transcribed. Interviews occurred a median of 26 (range: 3–170) days following a participant’s return. Those who consented and were within an appropriate time frame were interviewed a second time to check if anything had changed in the intervening period. First interviews lasted a median of 70 (35–123) min. Follow-up interviews lasted a median of 9 (5–13) min. Our questions (Appendix 1) broadly covered several predefined categories: effects of deployment on wellbeing, motivations for deploying, pre-deployment concerns, organisational processes, training, experiences while deployed, concerns regarding Ebola, family worries, support from others, and experiences on return. Interviews were semi-structured, with interviewers expected to cover all of the topics. However, flexibility was encouraged in terms of how an individual interview “flowed” (i.e., what topics where covered when). Interviewers also had freedom to probe further on any topics that appeared to be particularly important to the participant.

### Analysis

Within each interview, we categorised quotes as relating to the pre-, during-, or post-deployment period and then grouped quotes together that appeared to relate to the same overarching, predefined category (e.g., concerns pre-deployment). Within categories, we then grouped quotes together that tended to illustrate similar themes (e.g., lack of security in Africa) and created headings for themes and sub-themes that appeared to reflect the underlying issue. This process was iterative, with theme headings and structures being constantly reorganised as additional interview data were added. We assessed whether the structure and labelling of themes were stable (became “saturated”) during our analysis by assessing whether adding in quotes from additional transcripts substantively changed anything: in practice, data from the final interview transcripts changed very little. We elected not to interpret the data too deeply, and instead provided a relatively descriptive account of issues which appeared important to our participants (Braun & Clarke, 2006). Themes identified for only PHE or NGO staff are highlighted in the tables. In our results, we have only described the main themes that are most likely to be generalisable to future deployments, excluding some issues that appeared relatively less important (e.g., complaints about monotonous food) or issues that appeared specific to isolated mistakes or organisational idiosyncrasies (e.g., an isolated incident of confusion resulting in difficulties accessing water for one team).

We adopted several strategies to improve the credibility of the analysis (Shenton, 2004). As well as adopting well-established qualitative methods, being familiar with the organisations involved, and promoting honesty by reiterating the anonymity participants would have, two of us coded all of the data and came to a consensus on interpretation. Two others from our team independently coded data relating to organisational and social support. Comparison of their results and those of the main analysis revealed no substantial differences in interpretation. We also presented our findings to relevant charities and health agencies and ensured that all participants received a copy of our draft report. No substantive changes were required as a result of feedback.

### Role of the funding source

Our funders played no role in study design; data collection, analysis, or interpretation; writing the article; or decision to submit for publication.

## Results

We invited 104 PHE and 101 NGO staff to take part. Thirty PHE and 21 NGO personnel participated (median age 34 years (26–55 years); 28 women) in an initial interview. Ten PHE and eight NGO participants had a follow-up interview a median of 57 days later (range 31–93 days). The PHE group included some staff from related governmental organisations who deployed with PHE. The NGO group included 17 personnel from five charities, two NHS workers, one university researcher and one person who did not name their organisation. Our main themes are summarised in Tables 1–3. Themes are reported in the text below in italics.

**Table 1:** Main themes for pre-deployment period

Categories	Themes
Motivations	Uniqueness of opportunity, professional interest, excitement, desire to do something different, improving one’s CV, helping people, to see a tangible impact of your work, a sense of duty, the size of the public health need, employer general encouragement, employer facilitating taking time away.
Family interaction	Support from families, family did not understand, family worry, tension, guilt, worry about how the family would cope, concerns about the staff member posing a risk,^b^ wanting additional help from the organisation.
Training	Direct relevance, experienced trainers, opportunity to meet team members,^a^ too comprehensive, importance of a flexible attitude, practice with personal protective equipment.

a Theme appeared to be unique to PHE staff

b theme appeared to be unique to NGO staff.

**Table 2:** Main themes during deployment

Categories	Themes
Death and suffering	Impact on children, delivering bad news,^a^ meeting and seeing patients who subsequently die,^b^ learning the patient’s stories,^b^ connecting laboratory samples to a patient,^b^ guilt about not doing more,^b^ professional detachment, making an impact, being part of a global response, observing improvements, personal messages of thanks,^a^ normality among the community.
Contagion concerns	Moment of realisation of risk, hearing about workers contracting Ebola,^a^ trusting occupational procedures or personal protective equipment, proper procedures not followed, contagion outside the workplace, difficulties with no-touching rule, misinterpreting one’s own symptoms.
Organisational issues	Organisation operating well, concerned about protecting their safety and wellbeng, inconsistency between organisations,^b^ disputes between organisations,^b^ confusions about responsibility,^b^ unmet expectations,^b^ political pressure,^b^ bureaucracy and slow processes, working conditions.
Communicating with home	Beneficial for self, beneficial for families, not having the means to communicate, families used to having limited contact,^a^ not disclosing worrying information, providing reassurance, effect of bad news on families,^a^ exposure to additional worries from home.
Rest time	Adequate quantity, limited range of things to do, relaxing, feeling isolated, moral obligation to work.
Perceptions of the work	Fluctuating work load,^b^ clear role, autonomy, empowerment, gaining experience and skills,^b^ out of your depth, repetitive^b^, intense shifts, high expectations,^b^ boredom, frustration at not contributing more, suboptimal care,^a^ working in personal protective equipment in heat.^a^
Team support	Camaraderie, openness, teammates taking an interest, sharing a common purpose, learning from each other, not knowing your teammates,^b^ trusting a colleague’s proficiency. Team leaders: having their team’s best interests at heart, encouraging staff to have a say, approachable, competent, shielding staff, autocratic,^a^ added responsibility, worrying about their team.
Group interactions	Felt they were not a priority, lack of respect, not open to suggestions, different provisions, language barriers, perceiving some local staff as poorly motivated or trained.^a^

a Theme appeared to be unique to NGO staff

b theme appeared to be unique to PHE staff.

**Table 3:** Main themes after deployment

Categories	Themes
Support	Worry about being misdiagnosed with Ebola, screening reassuring, screening as patronising, unclear on what to expect on returning home, informal aspects of the follow-up, a sense of over being overlooked in follow-up,^a^ feeling that their contribution was appreciated by their organisation,^a^ option for time off.
Readjustment	Mundane day jobs, missing the deployment,^a^ sense of unfinished business, missing their team, friends, colleagues and family members as appreciative, supportive and interested, others could not understand,^b^ people being too interested, worried about other people’s reactions, being avoided by other people.
Overall impact	Personal and professional growth, increased confidence, new career options, networking, skills, experience, enjoyable experience, rewarding experience, psychological symptoms, dissatisfaction, doubt, or disappointment.

a Theme appeared to be unique to PHE staff

b theme appeared to be unique to NGO staff.

### Pre-deployment: motivations

Motivations for volunteering included: the *uniqueness* of the opportunity (“it doesn’t come round often that you’ve got the skills to help in a situation like this”), *professional interest* in the virus (“It was one of the most interesting organisms I studied”), for *excitement* or *desire to do something different*, *improving one’s CV* (“I knew how important it would be for my career progression”), *helping people*, to see a *tangible impact* of your work (“we mostly do research, so it’s hard to see immediate tangible benefit”), a *sense of duty*, and *the size of the public health need* (“I kind of felt this is my generation’s AIDS”). Many participants mentioned that support from their employer was important, either through *general encouragement* or by being *facilitated in taking time away* from their roles.

### Pre-deployment: family interaction

*Support from families* for the decision to go was helpful for many. The minority who reported that family members *did not understand* why they wanted to deploy found this more difficult (“they all pretty much thought I was mad”). *Family worry* was common and led to *tension* (“she said, ‘Why are you doing this, why are you leaving me?’”), *guilt*, and *worry about how the family would cope* (“you kind of feel like, it is your own fault and you don’t want to create that worry and anxiety for your friends and family”). In some instances, family worry reflected *concerns about the staff member posing a risk* on their return, an issue largely restricted to NGO participants. While many handled these issues well, some reported *wanting additional help from their organisation* (“maybe looking back on it I should’ve just given out someone at Public Health England’s number to talk?”).

### Pre-deployment: training

Feedback on pre-deployment training was largely positive, particularly its *direct relevance*. For PHE staff, the use of a mock laboratory to provide training was particularly praised (“it was like an exact replica of what the lab was going to be out there … and they did scenarios of the villages and people fainting. They even turned up the temperature to the max”). Obtaining tips from *experienced trainers* (“they had just so much advice on what to take, what you’d need, what you wouldn’t need, what to expect”) and the *opportunity to meet team members* was also valued. Negative feedback focused on the training being *too comprehensive*. The use of security training was found to be particularly unnecessary, being seen as an “over-reaction” by NGO employees and raising concerns in PHE staff. Many also suggested that training should emphasise the importance of a *flexible attitude* and, for NGO staff, *practice with personal protective equipment (PPE)*.

### During deployment: death and suffering

Death and suffering was by far the most common stressor that participants experienced. This appeared in many contexts, from laboratory personnel “wiping names off boards because people had died” to clinical staff seeing young children die. Several themes were apparent within this. The *impact on children* was particularly stressful. For NGO staff, *delivering bad news* was difficult, given the scale of the deaths (“we had a lady in and her father-in-law, her husband, her son, her brother, and her cousin all died”). *Meeting and seeing patients who subsequently died* was a new experience for many PHE staff (“in my [UK] hospital I’m in a building that’s several stories away from any of the patients … but [here] you can see patients die”). Patient contact led some PHE staff to *learn patients’ stories*, which could be “harrowing.” *Connecting laboratory samples to a patient* could also prove difficult. *Guilt about not doing more* was occasionally expressed by some PHE staff, who felt they could have either worked harder or intervened more in other ways (“Having people collapse outside the laboratory window and having to stand and watch”).

Despite this many staff maintained *professional detachment*, while positive aspects of *making an impact* and *being part of a global response effort* were commonly reported. The ability to *observe improvements* in patients was frequently described as positive by NGO personnel, while laboratory workers also reported satisfaction with their work (“it does really seem like you are saving lives out there every day”). This sense was heightened among clinical staff, many of whom felt moved by *personal messages of thanks* from members of the community. Equally, some participants reported expectations being confounded by a surprising *normality among the community*.

### During deployment: contagion concerns

Many participants described a sudden *moment of realisation* that the Ebola risk was real, linked to experiences such as hearing about a healthcare worker being medically evacuated or donning PPE for the first time. For NGO staff, *hearing about healthcare workers contracting Ebola* was particularly stressful. Most staff *trusted occupational procedures or PPE* to keep them safe in their workplace, but concern was higher when *proper procedures were not followed* due to mistakes, poor training or lack of equipment (“there were others putting us at risk not following the rules”). Concerns were also higher about *contagion outside of the workplace*, with a wide variety of possibly risky scenarios being described, ranging from concern about infection spreading from within treatment facilities (“we were slightly worried that there were others putting us at risk not following the rules”), to coming into contact with Ebola in local villages (“People would say ‘Oh my god, you bought a skirt in the market!’”), through to non-specific concerns (“In the first few days you were washing your hands frantically with alcohol every time you did something”). While staff trusted the advice they had been given to reduce risk (e.g., a “no-touching” rule), they were aware of the difficulty of enforcing these rules (“you’ve got loads of tiny kids … swinging off you, there’s not a lot you can do really”). *Difficulties with the no-touching rule* existed for a minority, for example through fear of being rude or being unable to comfort others. *Misinterpreting one’s own symptoms* as Ebola symptoms was common. Even when staff knew, rationally, that symptoms were almost certainly not Ebola-related, worry was still usual. This issue persisted for those interviewed several weeks after returning home (“I think you’re just so much more aware of every single symptom when you come back and you’ve been told to look out for things, so I think you’re just very conscious of everything”).

### During deployment: organisational issues

NGO staff described a reassuring sense that their *organisation was operating well* in supporting them (“people were working extremely hard around us invisibly”) and was *concerned about protecting their safety and wellbeing* (“I always felt that it was very well managed and that people were aware of what was happening and so they were trying their best to minimise any harm to us”). PHE staff hosted by NGOs reported *inconsistency between organisations* in terms of the quality of their organisation. For those working across organisations, *disputes between organisations* often caused frustration. Additionally, *confusions about responsibility* for looking after employees were described (“There were too many cooks […] there was no clear accounting line”). Perceived shortcomings that led to problems providing treatment were particularly criticised. In some cases, *unmet expectations* led to a loss of trust in organisations (“they didn’t quite know what they were doing”). Some PHE staff perceived *political pressure* to open laboratories on time and test sufficient numbers of patients. *Bureaucracy and slow processes* were frustrating for both groups. While many in both groups praised their *working conditions* (“It was really a pleasure to work in this lab,” “We were very resource rich”), instances of missing or damaged equipment were also reported.

### During deployment: communicating with home

Most participants described communication with friends and family as *beneficial for themselves* and *beneficial for their families* (“sometimes all you need is a phone-call home to make you feel better”). While many reported having multiple ways of contacting home, some found that *not having the means to communicate* presented difficulties, although some NGO staff reported that their *families were used to having limited contact* with them during deployments. Communication with home was not always straightforward in terms of what people discussed. For several participants, *not disclosing worrying information* or providing *reassurance* was an important consideration (“I hid most things from them, to be honest”). NGO participants specifically worried about the *effect of bad news on their families*, such as news that a UK healthcare worker had contracted Ebola. For a minority, contact with loved ones resulted in *exposure to additional worries from home* (“I thought, what have I done, [my baby at home’s] not sleeping well, she’s poorly…”).

### During deployment: rest time

Perceptions of the quantity and quality of rest time varied. While most felt that they had received an *adequate quantity* of time off work, some reported having few opportunities for time off (“we worked straight for 24 days”). Many participants described having a *limited range of things to do* on days off, being officially restricted to their hotels. This often felt frustrating (“you were essentially in a very nice prison”). Many described rest activities as simply *relaxing*. People were often not inclined to take rest days for many reasons including *feeling isolated* (“rather be at work where you’ve got social interaction”) and a sense of *moral obligation to work* (“don’t think I’d have felt right to be taking a day off”).

### During deployment: perceptions of the work

Laboratory workers described *fluctuating work-loads*, with periods of high intensity and inactivity. Clinical staff described a more consistent workload. Several positive aspects of the work were mentioned by both groups, including having a *clear role, autonomy, empowerment* and having the opportunity to *gain experience and skills*. Negative aspects included: a feeling of being *out of your depth*, though this usually dissipated quickly; the *repetitive* nature of the work, particularly for PHE personnel; *intense shifts* during busy periods; a perception of *high expectations* about their work among PHE staff; and *boredom* or *frustration at not contributing more* during slow periods (“you preferred to have lots of samples because the days just dragged”; “disappointing that we weren’t properly put to use, felt a bit guilty really that we were out there with not much to do”). For NGO staff, accepting that *suboptimal care* had to be offered due to a lack of resources, heat, language barriers, or safety procedures could be frustrating (“you knew if your Ebola patient was in the UK they would be getting so much more care and attention and facilities and resources”). The challenges of *working in PPE in heat* were also mentioned, including fears of vomiting inside PPE masks.

### During deployment: team support

Most participants described team *camaraderie* with good interpersonal relationships giving them a general sense of being supported (“I liked the group I was out with and that really, really helped”). Within this, several factors appeared to help participants cope with their deployment. First, many described a high level of *openness* within their team, with people being comfortable in asking for help with practical or emotional issues (“We were always very open to ‘if I’m doing anything wrong, tell me’”). Second, the reassuring sense of *teammates taking an interest* in your wellbeing was described in positive terms (“We were all looking out for each other, and I really appreciated that”). Third, *sharing a common purpose* was described as easing interactions and preventing competition or grievance (“it actually meant something to everyone, so there was none of that, ‘I do more than them’”). Participants reported that the mix of staff from different disciplines allowed staff to *learn from each other*. Conversely, n*ot knowing your teammates* was cited by several PHE staff as detracting from good teamwork. This appeared to be particularly problematic in a limited number of cases in which cliques formed among separate groups who had pre-existing connections. The importance of having to *trust a colleague’s proficiency* was also mentioned. As one participant said: “you were putting a lot of trust in people to do their job properly and likewise they were trusting you to do your job properly, because any sort of shortcuts or mistakes could have very serious consequences and I think once that trust was established, it’s really good in terms of the bond between everyone.”

Team leaders were generally described in positive terms, with most believing that they *had their team’s best interests at heart* (“she was in the lab every day, checking we were all OK”), e*ncouraged staff to have a say* and were *approachable* and *competent*. Perhaps the most important attribute of a good team leader was *shielding staff* from outside pressure, for example, by obtaining supplies, dealing with journalists, or negotiating bureaucracy. Among NGO staff, a feeling that the team leader was *autocratic* was less positive (“It was like being a junior house officer again and being frightened that your consultant might shout at you”). Although we interviewed relatively few team leaders, they noted that the *added responsibility* and *worrying about their team* could be stressful (“they’d do much longer shifts than everyone else and I think they felt a responsibility to always be there”). This was particularly true among NGO staff, many of whom found themselves responsible for large numbers of local staff (“we arrived at the same time as 30 national nursing staff … they had no formal qualifications and most had little, if any, training. So very quickly my role changed, morphed into preparing them for the wards”).

### During deployment: group interactions

Although harmonious working across organisations was common, instances where improvements could be made were described. PHE staff sometimes *felt they were not a priority* for their host organisation. Examples included being overlooked for accommodation, barred from using toilet facilities and not being provided with food or other resources, and generally being forgotten about or “left to our own devices.” Perceived *lack of respect* between groups was mentioned by multiple participants (“some of the staff were really rude to some of our staff at certain points when it got really stressful, you know, being really disrespectful or saying things out of line”), together with a perception that different agencies were *not open to suggestions* (“they just pawned us off”). *Different provisions* made for staff by employers were occasionally described as contentious. This theme included examples such as differences in the way pre-deployment vaccination was provided, provision of a mobile phone, policies on pay, and provision of information. *Language barriers* and, for some NGO staff, *perceiving some local staff as poorly motivated or trained* also detracted from good inter-group work.

### Post-deployment: support

Homecoming Ebola responders were screened for Ebola at the airport and received advice on how to monitor their physical health. While some *worried about being misdiagnosed with Ebola*, others found the *screening reassuring*. Still others dismissed *screening as* “*patronising*” or “a bit weird” given their own expertise. Being *unclear on what to expect on returning home* was a source of stress for several participants with apparent inconsistencies in the way groups of staff were treated and poor communication on what to expect being mentioned. Follow-up by employers after deployment was described as broadly adequate. Although few participants wanted formal support, several PHE personnel praised the *informal aspects of their follow-up*. Conversely, a small number of PHE staff described *a sense of being overlooked*, for example through not being screened or contacted by their occupational health department. *Feeling that their contribution was appreciated by their employer* was uplifting, although those who received a monetary award described it in non-committal terms. Being given an *option for time off* was widely described as helpful (“I spent it with my family, which was lovely. That was really nice. I think I was very tired and it relaxed me”).

### Post-deployment: readjustment

Many participants described a sense of deflation after their return from West Africa, described by one as “post-Ebola blues.” The same participant expanded on this: “I think quite a few of us are still feeling very, very sad about having left and very much rethinking life and we’re struggling to get back into normality and it’s all a bit of an effort, I think. It’s a bit of an effort to realise that it was such a life-changing experience and then it just immediately ended.”

Resuming a *mundane day job* and *“*struggling to care about emails” was a common difficulty for some who had “realised there’s a big, wide world [and] I could be doing other things.” Many PHE staff *missed the deployment* and both groups described a *sense of unfinished business* and *missing their team* (“I miss them a lot, I think that’s been the most difficult thing”). Interacting with other people was often challenging. Most participants described *friends, colleagues, and family members as appreciative, supportive, and interested*, although small numbers of NGO staff felt disconcerted that *others could not understand* what they had experienced. *People being too interested* posed challenges for a small number of participants who found themselves constantly recounting their stories (“after the first few days I just couldn’t face talking to anyone anymore”). Some participants *worried about other people’s reactions* and occasionally changed their behaviour to take the perceived concerns of others into account (“I didn’t want to put people in an awkward position where they felt they didn’t want me to be there”). These worries were often well-founded. Many participants reported *being avoided by other people* due to fears of contagion. This ranged from being a relatively minor issue (“A few other friends … didn’t exactly say they didn’t want to see me, but didn’t actively make arrangements to see me until I was past the 21 days”) to more upsetting incidents. While participants often made allowances for people with a poor understanding of Ebola, they were critical of those “who should know better.”

### Post-deployment: overall impact

Looking back, most participants reported positive effects from deployment. These included perceptions of *personal and professional growth* and *increased confidence* with people feeling a sense of achievement and pride in the work they had done (“there is a glow when people mention it”), feeling better equipped to take on future challenges (“I’m a bit more fearless now”) and describing a change in attitude (“I definitely appreciate what we have here [at home] a lot more”). Professional growth included thinking about *new career options*, *networking*, *skills*, and *experience*. People often described the work as having been *enjoyable* or *rewarding*. A minority of NGO participants, and even fewer PHE participants, reported *psychological symptoms*. These included reduced concentration, fatigue, anxiety, tearfulness, remembering upsetting incidents, avoiding discussion of the deployment, loss of motivation, nightmares, changes in appetite, and somatic symptoms such as headaches. More general negative effects of deployment were evident in a minority of participants, specifically *dissatisfaction*, and *doubt or disappointment* about the impact of one’s work.

## Discussion

This article presents evidence from the 2014/15 Ebola outbreak which is relevant to future deployment of staff to respond to global health threats. This includes how best to encourage staff to volunteer for such work. While most participants cited moral reasons for volunteering, desire for personal benefits such as for excitement or developing skills was common. Previous studies on volunteering for the Ebola response have not explored responders’ personal goals (Rexroth et al., 2015; Turtle et al., 2015). Communicating with staff about the opportunities that exist to further personal goals may improve future recruitment efforts.

We identified that thoroughly preparing responders for their work was viewed as essential. Reassuringly, participants broadly praised their training as realistic and relevant. Future training should be viewed as opportunities to foster camaraderie between teammates prior to deployment.

Supporting family members before and during the deployment was also viewed as important. As in previous outbreaks (Hall, Hall, & Chapman, 2008) multiple family-related stressors were described. The importance of having one’s family looked after has been raised before by crisis responders (Bakhshi et al., 2014) and shown to affect the mental health of military personnel (Mulligan et al., 2012). Research on how best to support family members of crisis responders is warranted.

As expected (Greenberg et al., 2015; Hall et al., 2008; Hewlett & Hewlett, 2005), concerns about contagion and difficulties dealing with death and suffering were common. Preparatory training, especially for inexperienced staff, should cover mechanisms for coping with these issues. However, our results also highlighted many uplifting aspects of deployment such as seeing improvements in patients and receiving thanks from locals. Happenstance or organisational policies can mean that some staff miss out on these experiences; team leaders should ensure that no-one misses out entirely.

Less expected were the reports of inter-organisational difficulties which were reported as being especially difficult to deal with. Improving the integration of different organisations should be prioritised before any future deployment.

Although concern has been expressed about the possible mental health consequences of responding to the Ebola crisis (Greenberg et al., 2015), few participants described symptoms indicative of a formal psychiatric disorder. Other problems such as dissatisfaction, and doubt or disappointments were more common. It was also evident that some staff felt they were not followed up after deployment. Formalised follow-up should allow for early detection and resolution of mental health difficulties and other problems which are common issues following crisis work (Bakhshi et al., 2014), and may sustain motivation to carry out similar work in future.

A more specific problem is avoidance of responders by other people. While most participants were tolerant of this, frustration was directed at people or organisations “who should know better.” This issue is not unique to our sample (McCarthy, 2014) nor to Ebola (De Jarlais, Galea, Tracey, Tross, & Vlahov, 2008). In future, employers could provide information to those who might be concerned in order to improve the homecoming experiences of staff.

While broadly similar themes appeared to capture the experiences of both the PHE and NGO groups, we did observe some differences between them. Many of these differences can be ascribed to the difference between the two groups in terms of levels of experience working during a humanitarian crisis. For example, while participants from both groups would have witnessed death, heard patients’ stories, or had moments where they felt they could have done more during their work in West Africa, these themes had substantially greater resonance for the generally more inexperienced PHE staff. Other differences may reflect the different tasks that the two groups engaged in. For example, the clinical work of NGO staff meant that the stressful role of delivering bad news fell solely to them, as did the uplifting experience of being personally thanked by members of the community. These differences highlight the importance of tailoring future training to the specific experiences and roles of different teams of responders.

Several caveats should be borne in mind for this work. First, selection bias may have led to personnel who considered that they had something interesting to contribute being more likely to take part. Second, while qualitative methods allowed us to identify issues that may otherwise have been overlooked, we cannot say how prevalent these issues were among all PHE or NGO personnel who went to West Africa to help with the Ebola response: a quantitative study would be required to provide a useful estimate of this. Third, while we see no reason why our results would not generalise to staff from other organisations, we cannot rule out the possibility that differences in organisational culture or types of prior experience may exist, meaning that some of the themes we identified are not relevant, while other relevant themes may exist that we did not identify. The existence of differences between our PHE and NGO participants confirms that such differences can exist.

Overall, our results suggest that while responders were proud of the work they did in West Africa, greater attention to training, monitoring, and supporting staff members and their families is warranted. Issues such as the importance of team-building prior to deployment, inter-organisational difficulties, family concerns, post-deployment loss of motivation, and inequitable access to uplifting experience can and should be anticipated before future deployments.

## Appendix 1: Interview schedule for participants interviewed in two stages

Instructions to interviewer on where to probe further are given in italics. Other instructions to interviewer have been removed for clarity.

***First stage of interview***

Before you were deployed to West Africa, had you ever done any crisis or overseas work? What was it? *Probe for crisis response, even if in UK*.

Did that experience help you or hinder you at all this time round?

And did you have any specific training or preparation before you went out? Can you tell me what you had?

In retrospect, how helpful was it? Is there anything that should be done to make it better?

You must have had some expectations of what things would be like before you went. Did your experiences match your expectations?

What were your biggest worries or concerns before you went out?

Were you worried about your own safety before you went? Did you have any concerns for your friends or family back at home?

Before you went, did your family have any concerns or misgivings? What were they? How did you deal with that?

Was there any specific information or preparation given for families? Do you think it helped/do you think there should have been?

How well do you think things were organised by your employer? Was there any aspect of the way work was run that made things difficult or stressful for you? Or that made life easier or more positive for you?

In terms of the resources or planning or infrastructure that you had available, do think there was anything there that made life especially difficult or easier for you? *Probe for equipment to do job safely (protective kit) and also effectively. Also probe for living conditions/food/recreation exercise kit*.

How long were you deployed for? Was that what you expected? *If no:* What did you think about that?

And how much notice did you get before you deployed? Was that enough?

How much work was there to do? Did you have enough time to deal with everything? Did it get boring at times? How did you cope with that?

How much choice did you have about what to do and how to do it?

Was it always clear what you were meant to be doing? If not how was the uncertainty dealt with (e.g., good boss or just left to decide themselves).

How did things go in terms of communication within your organisation—did you feel you understood what was going on and why?

Do you think people at your work took/would have taken any feedback from you on board? First of all your colleagues and secondly your line managers?

How about communication back home—did you have access to phones or the internet? Was it good enough?

There were lots of different teams trying to deal with outbreak: PHE deployed and in the UK, NGOs, local responders. How did it go with all those teams trying to work together?

Was there anything else in terms of how the operation was run that you found stressful or frustrating? Or particular helpful?

Did you find it easy to take time off and rest while you were out there? *Probe for any barriers to taking rest*

On your time off, what options did people have for unwinding? And what were you personally doing in your time off?

If you had wanted to, do you feel you could have spoken to one of your colleagues about how you were feeling? What about your line manager?

Did you feel that your co-workers were looking out for you/checking how you were coping? Did you do similarly for them?

What about people further up the chain in your organisation? Did you feel that your line manager was taking an interest in your wellbeing? Would you have felt comfortable talking to them if you had been feeling under strain or upset?

Can I ask the same thing about your family or loved ones outside of work? Where they concerned about you? How did you discuss their concerns with them? How often could you communicate with them and how did you?

We’ve heard people say before that dealing family can be tricky in situations like this—sometimes they don’t want you to go and there can be tensions. Did you experience anything like that? Where there things about your deployment you did not discuss with them?

The fact that the outbreak involved Ebola. Did that worry you at all or affect you? Why?

In your job, was there ever a risk that you might be directly exposed to Ebola (e.g., near misses)? What did you think about that?

Did you ever worry that you might be exposed?

What about others you were working with?

Did you ever come into contact with people who were suffering with Ebola, or their relatives? How did you find that experience?

Did you interact much with the local community while you were there? How did they react to you? What effects did the interaction have you on? *We are looking for hostility or welcoming/appreciative interactions*.

And you were in a place where many people had already died—was that apparent/did it affect you personally?

And aside from Ebola, there all sorts of other things more generally about working in West Africa that people can find surprising, enjoyable, or stressful. Was there anything about working there that you found stressful or uplifting. *Probe for poverty, corruption, environment, seeing new places/culture, exposure to other hazards (*e.g., *malaria), culture clash, climate*.

Another somewhat different aspect about this deployment is that you were in the public eye. Politicians were interested, the media were interested. Were you conscious of that at the time? Did it have any impact on how you did you job or your wellbeing?

People have lots of different reasons for doing this work. Why did you go? Probe for excitement, wanting to help, any sense of compulsion/expectation?

Did you achieve that, for example, excitement/potential career advancement etc?

Overall, do you think the response by your organisation in West Africa has helped people out there? How about your team? And what about you personally—do you feel YOU helped people out there? Why/why not? Did it feel like you were helping at the time?

We are interested in whether your work in Sierra Leone has had any longer term impact for you. First of all, would you say the deployment has had any effect on your career prospects?

What about the way you see yourself, or the world? *Prompt for esteem, negative or positive views*.

Some people can find deployments like this affect their physical or mental health. Have you noticed that your health has been affected at all? *Probe for any effects on sleep/stress*.

Would you do it again?

We are interested in what challenges or opportunities you think might lie ahead for you and also your family, now that you have returned from your deployment. Can I break it down into three areas and ask what you think about each?

First of all what do you think the challenges or opportunities are likely to be for you at work in the short term? What about in the longer term? Do you think your deployment will affect your job prospects in the future?

Second what do you think the challenges or opportunities are likely to be at home or in your social life in the short term? Do you think there will be any longer term effects especially over the next few months?

Third what do you think the challenges or opportunities are likely to be for your health in the short term? What about over the next few months? What might be helpful to improve your health and wellbeing over the months ahead?

And can I ask what you think your organisation should be currently doing to help with your homecoming and over the next few weeks to help you? *Probe for follow-up, pay, time off, help with family*.

Overall, do you think this deployment will have any substantial effect/impact on your life in the longer term.

That is all the questions I had for you. But before we turn the recorder off, is there anything else you want to say about your deployment or anything else?

***Second stage of interview***

Thanks for allowing us to speak to you again. Really we just wanted to check with you have you have been getting on since we last spoke. How have things been?

We are interested in whether your work in Sierra Leone has had any longer term impact for you. First of all, would you say the deployment has had any effect on your career prospects?

What about the way you see yourself, or the world? *Prompt for esteem, negative, or positive views*

Some people can find deployments like this affect their physical or mental health. Have you noticed that your health has been affected at all? *Probe for any effects on sleep/stress*

How do you feel now about your work after the disaster? Rewarding? Unrewarding? Upsetting? Rewarding?

Would you do it again?

And how have things been at home or with your social life?

How have things been at work?

How have things been with your health?

And did you get any support or follow-up from your employer? Or pay/time off?

When we spoke last, you mentioned thinking that X might be a challenge/opportunity. How did things turn out?

That is all the questions I had for you. But before we turn the recorder off, is there anything else you want to say about your deployment or anything else?
